# Mental health resilience in offspring of depressed parents: a systematic literature review protocol

**DOI:** 10.1186/s13643-022-02056-6

**Published:** 2022-09-05

**Authors:** Eglė Padaigaitė, Jessica Mayumi Maruyama, Gemma Hammerton, Frances Rice, Stephan Collishaw

**Affiliations:** 1 Wolfson Centre for Young People’s Mental Health, Section of Child and Adolescent Psychiatry, Division of Psychological Medicine and Clinical Neurosciences, Cardiff University, Hadyn Ellis Building, Maindy Road, Cardiff, CF24 4HQ UK; 2 Departamento de Medicina Preventiva, Faculdade de Medicina FMUSP, Universidade de São Paulo, São Paulo, Brazil; 3 Population Health Sciences, Bristol Medical School, University of Bristol, Bristol, UK; 4 Medical Research Council Integrative Epidemiology Unit, University of Bristol, Bristol, UK

**Keywords:** Mental Health, Resilience, Offspring, Parents, Depression, Systematic review

## Abstract

**Background:**

Parental depression is associated with a range of mental health conditions and other difficulties in the offspring. Nevertheless, some offspring exposed to parental depression do not develop mental health problems, indicating the presence of protective factors that may buffer parental depression-related risk effects. However, evidence of protective factors that might explain good sustained mental health in offspring of depressed parents is limited and systematic synthesis of these factors is still needed. Therefore, as far as we are aware, this will be the first systematic review that will identify parent, family, child, social, and lifestyle factors associated with mental health resilience in offspring of depressed parents, examine evidence for sex-, developmental stage-, and outcome-specific factors and define mental health resilience in the parental depression context.

**Methods:**

This protocol has been developed according to the PRISMA-P guidelines. Electronic searches will be performed for articles published up to 2022 in PsycINFO, Embase, MEDLINE, Web of Science Core Collection, and Cochrane Library. Two reviewers will independently screen titles/abstracts and full-texts against eligibility criteria, extract the data, and assess the overall quality of evidence. Both observational and RCT studies will be eligible for inclusion if they report offspring mental health resilience/outcome and depressive symptoms or depressive disorder in at least one of the parents/caregivers. Risk of bias will be assessed using The Joanna Briggs Institute (JBI) critical appraisal checklists and The Revised Cochrane risk of bias tool for randomised trials (RoB 2). It is expected that studies will be heterogeneous; therefore, meta-analysis will not be attempted. Studies will be systematically retrieved and collated using numerical, graphical, tabular, and narrative summaries and grouped by their design, scope, or overall quality. Further sub-group analyses will be performed to examine sex-, developmental stage-, and outcome-specific protective factors.

**Discussion:**

The proposed systematic review will be the first to summarise and critically assess quality and strength of evidence of protective factors associated with mental health resilience in offspring of depressed parents. Directions and effect sizes of the protective factors will be discussed as well as differences between the studies, their limitations, and research gaps and future directions. Strengths and limitations of the proposed systematic review will be also discussed. The proposed systematic review findings are expected to help better understand mental health resilience and identify targets for evidence-based prevention and intervention strategies for those in need.

**Systematic review registration:**

A previous version of this systematic review protocol has been registered in the International Prospective Register of Systematic Reviews (PROSPERO) database (www.crd.york.ac.uk/PROSPERO, CRD42021229955).

**Supplementary Information:**

The online version contains supplementary material available at 10.1186/s13643-022-02056-6.

## Background

Adult depressive disorders and symptoms (depression) are common mental health conditions characterised by mood, somatic and cognitive symptoms that significantly affect an individual’s daily functioning, including core features of anhedonia, loss of interest/pleasure, and decreased energy [1]. Depression affects more than 264 million people worldwide and has been one of the leading causes of disability adjusted life years [2]. Depression has not only been associated with higher risk of dying by suicide [3], substance abuse [4], development of medical conditions [5, 6], and impaired social functioning [7] in individuals suffering from this condition but also with difficulties in social relationships, education, and mental health problems in their offspring [8, 9]. Considering that up to one in five children aged 0–16 years old in the UK are exposed to parental depression and these numbers continue to rise [10], identification of factors that could improve the lives of depressed parents and their offspring remains an issue of a high public health importance [11].

Offspring of depressed parents are almost four times more likely to meet diagnostic criteria for depression compared to those of parents without a history of mental health conditions [12]. Interestingly, elevated risk of psychiatric disorders in this population is not limited to depression, and offspring of depressed parents are also more likely to suffer from a range of other mental health conditions, such as anxiety, substance use, conduct disorders, and attention deficit hyperactivity disorder (ADHD) [13, 14]. As demonstrated by Tully et al. [15], both inherited and environmental processes may be involved in the intergenerational transmission of psychopathology.

A growing body of evidence indicates that risk of psychopathology in a child may vary according to the severity and nature of parental depression. Parental depression characteristics such as severity, recurrence, and chronicity [16] and comorbid parental mental health conditions [17] have been shown to be associated with offspring psychopathology. Furthermore, depression is commonly accompanied by exposure to a range of negative life events and an increased risk of interpersonal difficulties such as marital discord [18] or parenting difficulties [19]. It has been suggested that exposure to social adversities associated with parental mental health problems may be even more influential than the parental depression itself [20].

The effect of parental depression on child’s psychopathology may be also influenced by the sex of the offspring and the parent [21]. Although the majority of previously conducted studies included depressed mothers only, some studies also examined the role of fathers in shaping offspring mental health outcomes. Previous studies reported both the presence [22] and absence [15] of association or even the ability of positive father-child interaction to buffer maternal depression effects on offspring psychopathology [23, 24]. Therefore, it is still unclear whether parental depression effects are stronger for same-sex parent-child or mixed-sex parent-child dyads [25]. Inconsistent results could be potentially explained by ‘assortative mating’ [26] — a tendency for people to choose romantic partners who show similarities in personality or life experiences — that further complicates complex processes behind the intergenerational transmission of psychopathology.

Although offspring of depressed parents are at increased risk for psychopathology, some do not develop mental health difficulties. A longitudinal study by Collishaw et al. [27] revealed that one in five adolescent offspring exposed to parental depression demonstrates mental health resilience, while Rutter et al. [28] found that about one third of those who develop mental health difficulties do so only temporarily. Mental health resilience — relative resistance to psychopathology despite serious risk exposure [29] — observed in offspring of depressed parents indicates the presence of protective factors that may buffer parental depression-related risk effects. Identification of these protective factors and an understanding of the processes through which individuals exposed to parental depression overcome experienced adversities will lead to a better understanding of mental health resilience and what distinguishes adaptive and maladaptive trajectories and also help to identify targets for evidence-based prevention and intervention strategies for those in need [30].

Despite being identified as a priority [11], evidence of protective factors that might explain mental health resilience in offspring of depressed parents is limited and systematic synthesis of these factors is still needed. Therefore, to our knowledge, the proposed systematic literature review will be the first one to examine factors associated with mental health resilience in offspring of depressed parents.

## Methods

### Design and registration

The protocol for proposed systematic review was developed according to the Preferred Reporting Items for Systematic Review and Meta-Analyses Protocols (PRISMA-P) guidelines [31]. For completed PRISMA-P checklist, see Additional file 1. A previous version of this protocol has been registered in the International Prospective Register of Systematic Reviews (PROSPERO) database (www.crd.york.ac.uk/PROSPERO, CRD42021229955). The current protocol is a more detailed version of the one registered in PROSPERO. In response to the external review, the current protocol has been revised, so it includes articles up to 2022 and excludes the forward and backward chaining approach step; data extraction and risk of bias assessment are independently performed by two reviewers, and more details are added on eligibility criteria and data synthesis of expected results.

### Objectives

Following the PEO (i.e. Population, Exposure, Outcome) format for systematic reviews of association (aetiology) [32], the primary aim of the proposed systematic literature review will be to systematically search and narratively synthesise studies examining parent, family, child, social, and lifestyle factors (exposure) associated with mental health resilience (outcome) in offspring of depressed parents (population). As a secondary aim, we will examine if there is evidence to support sex-, developmental stage-, and outcome-specific factors. Finally, we will synthesise information from previous research on how mental health resilience was conceptually and operationally defined, which offspring outcome domains were examined to define resilience, and over what time period.

### Eligibility criteria

#### Study design

Only primary research studies published in peer-reviewed journals will be included in the proposed systematic review. Both observational studies (e.g. high risk and population cohorts, case-control or cross-sectional) and randomised controlled trials (RCT) will be included if they report effect sizes of examined protective factors and child’s mental health outcomes. Secondary research (e.g. book chapters, reviews, letters, editorials, or commentaries), grey literature, qualitative (i.e. using non-numerical data) studies, systematic reviews, meta-analyses, abstracts, or conference proceedings will not be eligible for inclusion.

#### Population

The proposed systematic review will consider studies examining protective factors in offspring of depressed parents. Offspring in the study can be of any age (i.e. from childbirth to adulthood), but to be eligible for inclusion, at least one of the child’s parents/caregivers in the study has to meet clinical or research International Classification of Diseases (ICD) or Diagnostic and Statistical Manual of Mental Disorders (DSM) criteria for a depressive disorder, receive treatment for depressive disorder, or report depressive symptoms. Studies, including parents/caregivers with mental health comorbid conditions (e.g. anxiety), will be considered if parents also report depressive disorder/symptoms. Therefore, studies examining mental health conditions more broadly (i.e. presence of any mental health disorder) will be excluded. Studies reporting parent’s depressive symptoms in other specific populations (e.g. depressed parents of autistic offspring, depressed parent of offspring with cancer) or contexts (e.g. studies in the context of pandemics) will also be excluded since it would complicate comparisons of studies and limit generalisability of the results.

#### Exposure

Prospective and retrospective cohorts, case-control and cross-sectional studies examining parent (e.g. parental depression characteristics, parenting), family (e.g. socioeconomic status, parent-child relationships), child (e.g. biological and cognitive factors), social (e.g. peer relationships), and lifestyle (e.g. exercise) factors at any time (i.e. from childbirth to adulthood) will be eligible for inclusion. Information on protective factors can be obtained through registers, medical records, clinical assessments or offspring-, parent-, or teacher-reported questionnaires. Both high risk cohorts examining protective factors as predictors and population cohorts examining protective factors as moderators between parental depression and child’s mental health outcomes will be included.

#### Outcome

Since mental health resilience can be conceptually and operationally defined in various ways and research still lacks a universally accepted definition [33], the proposed systematic review will not exclude studies based on the definition used. Therefore, studies reporting offspring’s mental health outcome or using varying definitions of mental health resilience, such as the absence of psychopathology, positive adjustment/competence, or better than expected mental health outcomes, will be eligible for inclusion. In eligible studies, information on offspring’s mental health outcomes can be obtained through registers, medical records, clinical interviews or assessments, or using offspring-, parent-, or teacher-reported validated instruments. Since the proposed systematic review will focus on common mental health and behavioural problems, studies reporting offspring’s psychiatric diagnoses (e.g. major depressive disorder) and symptoms (e.g. anxiety symptoms), internalising, externalising, emotional, or behavioural problems will be eligible for inclusion. Studies using brain areas associated with mental health conditions as a primary outcome and studies examining suicidal behaviour, substance use, smoking behaviours, or neurodevelopmental conditions (e.g. autism, ADHD, intellectual disability) will be excluded.

#### Language and publication year

Only studies published in English will be eligible for inclusion. No restrictions on publication year will be applied.

### Search strategy

Electronic searches will be performed by searching titles, abstracts, keywords, and subheadings for articles published up to 2022 in PsycINFO, Embase, MEDLINE, Web of Science Core Collection, and Cochrane Library. The first three databases will be accessed using the Ovid platform, while Clarivate will be used to access Web of Science Core Collection. No filters will be applied during the search.

To maximise the chances of identifying relevant studies, search strings will be constructed combining index words — broader concepts that also capture all narrower terms associated with these (e.g. Emtree words in Embase) – and terms organised in five key categories: parents/caregivers, depression, offspring, protective factors/mental health resilience, and exclusion terms. The combination of index words and terms that will be used in electronic searches is presented in Table 1, while a draft search strategy for one of the databases is presented in Table 2. Within each category, all related terms and index words will be combined with the operator OR. Then, the first four categories will be combined with the operator AND. Finally, exclusion terms will be added using the operator NOT to eliminate secondary research reports, conference proceedings, qualitative studies, reviews, and meta-analyses from relevant studies identified (for more details see Additional file 3 where the full search strategy for each database is presented). Terms and subheadings for the electronic searches were chosen on the basis of results of an initial scoping review, search terms used in previous narrative reviews on this topic, consultation with the university librarian, and researchers with expertise in developmental psychopathology, mental health resilience, and systematic literature reviews [34].

**Table 1:** Subject headings and search terms that will be used in the proposed systematic review

Key concept	Index words		Terms
**Parents/caregivers**	caregiver OR caregivers OR parent OR parents	OR	caregiver* OR parent* OR maternal OR paternal OR mother* OR father*
AND			
**Depression**	depression OR mood disorder OR mood disorders OR affective disorders OR major depression	OR	depress* OR affective disorder* OR mood disorder*
AND			
**Offspring**	child OR offspring	OR	offspring* OR child* OR son* OR daughter*
AND			
**Protective factors/mental health resilience**	psychological resilience OR resilience, psychological OR resilience (psychological) OR protective factors	OR	resilien* OR protect* OR buffer* OR mitigat* risk OR optimi* outcome* OR adapt* functioning OR thriv* OR positive adaptation
NOT			
**Exclusion terms**	“review” OR “literature review” OR “systematic review” OR metaanalysis OR meta-analysis OR qualitative methods OR qualitative research OR conference paper OR congresses	OR	review* OR comment* OR letter OR meta-analysis OR metaanalysis OR editorial OR conference publication OR qualitative

**Table 2:** Draft search strategy for one of the databases

Database	Terms
Ovid MEDLINE	1 exp Parents/ 2 exp Caregivers/ 3 exp Mood Disorders/ 4 exp Depression/ 5 exp Child/ 6 exp Resilience, Psychological/ 7 exp Protective Factors/ 8 exp “Systematic Review”/ 9 exp “Review”/ 10 exp Meta-Analysis/ 11 exp Qualitative Research/ 12 (caregiver* or parent* or maternal or paternal or mother* or father*).ab,kw,ot,ti. 13 (depress* or (affective adj2 disorder*) or (mood adj2 disorder*)).ab,kw,ot,ti. 14 (offspring* or child* or son* or daughter*).ab,kw,ot,ti. 15 (resilien* or protect* or buffer* or (mitigat* adj2 risk) or (optimi* adj2 outcome*) or (adapt* adj2 functioning) or thriv* or (positive adj2 adapt*)).ab,kw,ot,ti. 16 (review* or comment* or letter or metaanalysis or meta-analysis or (meta adj2 analysis) or editorial or (conference adj2 publication) or qualitative).ab,kw,ot,ti. 17 1 or 2 or 12 18 3 or 4 or 13 19 5 or 14 20 6 or 7 or 15 21 8 or 9 or 10 or 11 or 16 22 17 and 18 and 19 and 20 23 22 not 21

Pilot testing of the search strategy was performed prior to registering the protocol to identify the utility of selected subject headings, terms, and their combinations. Ten publications relevant to mental health resilience, including literature reviews and qualitative studies, were identified prior to the pilot search. Then, electronic searches were performed to examine if the search strategy used captured relevant publications identified and excluded those that employ research designs not eligible for inclusion (i.e. qualitative studies, systematic reviews, and meta-analyses). For selected publications and pilot search results, see Additional file 2.

### Screening procedure

All search results will be exported from the database and imported into the reference management software EndNote™ and automatically deduplicated by matching different combinations of author, title, and year of study. Then, to catch any outstanding duplicates, further manual deduplication will be performed. Following deduplication, references will be imported into systematic literature review software Rayyan [35] that will be used for title/abstract and full-text screening, documentation of screening progress, and reasons for exclusion. First, titles and abstracts will be screened to identify publications for full-text retrieval. If, at this stage, reviewers are unsure about the eligibility of the study, it will be included in the full-text screening stage. Then, full texts of potentially eligible studies will be assessed against previously mentioned eligibility criteria. Discrepancies between reviewers, if any, will be resolved during consensus meetings with a senior researcher (SC). The literature search and deduplication will be performed by the first reviewer only (EP), while title/abstract and full-text screening of studies identified by electronic searchers will be independently undertaken by both first and second (JMM) reviewer. Reviewers will be blinded to the other’s decisions during this process.

### Data extraction

Two reviewers will independently extract the data from relevant studies and discrepancies will be resolved during meetings with a senior researcher (SC). A data extraction form will be developed following Preferred Reporting Items for Systematic Review and Meta-Analyses (PRISMA) [31] and Cochrane [36] guidelines and will be piloted and revised to facilitate the extraction before further use. Extracted data will be compiled in an Excel sheet. The data extraction form will include information on study characteristics (i.e. authors, title, year, study type and design, country, sample size, number of females and males, and study aims), population characteristics (i.e. depressed parent’s age and sex, type and characteristics of parental depressive disorder, comorbid conditions, and assessment and diagnostic instruments used), exposure (i.e. type of protective factor, how it was assessed, unadjusted and adjusted effect size, confidence interval, *p* value, and confounders (if any) used in the model), outcome (i.e. type of outcome, how it was assessed, offspring age at assessment, and definition of mental health resilience (if appropriate)), and information on comparison group (if any). Additionally, for RCTs, baseline and intervention characteristics (i.e. type of intervention, timing, duration, and delivery) will be extracted. Main study findings, limitations, and risk of bias assessment outcome will also be documented in the data extraction form.

### Risk of bias assessment

The risk of bias assessment will be independently performed by both reviewers and Cohen’s Kappa coefficient will be calculated to assess inter-rater agreement between two reviewers. For observational studies, Joanna Briggs Institute (JBI) critical appraisal checklists for cohort, case-control, and cross-sectional studies will be used [37], and, in line with recommendations, no studies will be excluded based on the risk of bias assessment outcome [38]. This critical appraisal tool considers comparability of groups, appropriateness of exposure and outcome assessments, identification and handling of confounding factors, and appropriateness of statistical analyses used. Each domain will be evaluated as being at a high, low, or unclear risk of bias or by selecting a not applicable option.

The Revised Cochrane risk of bias tool for randomised trials (RoB 2) will be used for RCTs [39]. The risk of bias arising from the randomisation process, deviations from the intended intervention, missing outcome data, measurement of outcome data, and selection of the reported results will be judged by selecting one of five options (Yes; Probably Yes; Probably No; No; No Information).

### Data synthesis

Due to expected heterogeneity across studies in terms of outcomes and protective factors examined, study designs used, and effect sizes reported, meta-analysis will not be attempted. However, if appropriate, information will be systematically retrieved and collated using numerical, graphical, tabular, and narrative summaries. First, we will provide a descriptive numerical and tabular summary of total number of included studies and will describe their key characteristics, such as country, year of publication, study design, sample size, and the risk of bias assessment outcomes. Then, to address the first research question, all examined protective factors will be collated into categories (e.g. parent, family, child, social, and lifestyle) and presented in a table or graph. Considering that depressive symptoms may reflect general distress rather than depressive disorder [40, 41], the results of studies reporting depressive symptoms and depressive disorders in parents/caregivers will be reported separately. If a large number of studies are identified, articles might be also summarised based on their overall quality (e.g. high; moderate; low). If appropriate, further sub-group syntheses will be performed to examine sex-, developmental stage-, and outcome-specific protective factors. Additional quantitative data synthesis approaches, such as weighted effect sizes or funnel plots, will be adopted if appropriate. Finally, a summary of mental health resilience definitions used in the studies will be provided.

## Discussion

To our knowledge, the proposed systematic literature review will be the first one to assess the quality and strength of evidence of protective factors associated with mental health resilience in offspring of depressed parents. Directions and effect sizes for the protective factors will be discussed as well as differences between the studies, their limitations, and research gaps and future directions. Strengths and limitations of the proposed systematic review will be also discussed. Key strengths of the proposed systematic review are a rigorous and transparent approach for identification and retrieval of existing literature on the topic documented in the current protocol and the development of a search strategy in consultation with the medical librarian and experts in the field. Limitations include language restriction to English and grey literature exclusion which could lead to omission of some relevant publications and limit the generalizability of the results. However, if possible, in the proposed systematic review, we will aim to address potential publication bias with funnel plots. The proposed systematic review findings are expected to increase understanding of mental health resilience and help to identify targets for evidence-based prevention and intervention strategies for those in need.

## Supplementary Information

**Additional file 1.** PRISMA-P (Preferred Reporting Items for Systematic Review and Meta-Analysis Protocols) checklist: recommended items to address in a systematic review protocol

**Additional file 2.** Selected publications for pilot search and pilot search results

**Additional file 3.** Full search strategy for each database

## Data Availability

Not applicable.

## Abbreviations

ADHD: Attention deficit hyperactivity disorder
PRISMA-P: Preferred Reporting Items for Systematic Review and Meta-Analyses Protocols
PROSPERO: International Prospective Register of Systematic Reviews
PEO: Systematic review question format outlining population or group at risk, exposure, and outcome
RCT: Randomised controlled trial
ICD: International Classification of Diseases
DSM: Diagnostic and Statistical Manual of Mental Disorders
PRISMA: Preferred Reporting Items for Systematic Review and Meta-Analyses
RoB 2: Revised Cochrane risk of bias tool for randomised trials
