# Supplementary material for: Mental health resilience in offspring of depressed parents: a systematic literature review protocol
Source: Syst Rev. 2022 Sep 5;11:190. doi: 10.1186/s13643-022-02056-6 (PMC9446554; doi:10.1186/s13643-022-02056-6)
Supplement: Supplementary file 2 — Additional file 2. Selected publications for pilot search and pilot search results [file 13643_2022_2056_MOESM2_ESM.pdf]

Selected publications for pilot search and pilot search results

**Publication**	**Whether it should be identified (based on eligibility criteria)?**	**Whether it has been identified during the search?**
Collishaw, S., Hammerton, G., Mahedy, L., Sellers, R., Owen, M. J., Craddock, N., ... & Thapar, A. (2016). Mental health resilience in the adolescent offspring of parents with depression: a prospective longitudinal study. *The Lancet Psychiatry*, *3*(1), 49-57.	Yes	Yes
Pargas, R. C. M., Brennan, P. A., Hammen, C., & Le Brocque, R. (2010). Resilience to maternal depression in young adulthood. *Developmental psychology*, *46*(4), 805.	Yes	Yes
Downey, G., & Coyne, J. C. (1990). Children of depressed parents: an integrative review. *Psychological bulletin*, *108*(1), 50.	No	No
Goodman, S. H. (1987). Emory University project on children of disturbed parents. *Schizophrenia Bulletin*, *13*(3), 411-423.	Yes	Yes

Luthar, S. S., & Sexton, C. C. (2007). Maternal drug abuse versus maternal depression: Vulnerability and resilience among school-age and adolescent offspring. *Development and Psychopathology*, *19*(1), 205.	Yes	Yes
Vakrat, A., Apter-Levy, Y., & Feldman, R. (2018). Sensitive fathering buffers the effects of chronic maternal depression on child psychopathology. *Child Psychiatry & Human Development*, 49(5), 779-785.	Yes	Yes
Davydov, D. M., Stewart, R., Ritchie, K., & Chaudieu, I. (2010). Resilience and mental health. *Clinical psychology review*, *30*(5), 479-495.	No	No
Focht-Birkerts, L., & Beardslee, W. R. (2000). A child's experience of parental depression: Encouraging relational resilience in families with affective illness. *Family process*, 39(4), 417-434.	No	Yes
Jaser, S. S., Champion, J. E., Dharamsi, K. R., Riesing, M. M., & Compas, B. E. (2011). Coping and positive affect in adolescents of mothers with and without a history of depression. *Journal of Child and Family Studies*, *20*(3), 353-360.	Yes	Yes

Priel, A., Djalovski, A., Zagoory-Sharon, O., & Feldman, R. (2019). Maternal depression impacts child psychopathology across the first decade of life: Oxytocin and synchrony as markers of resilience. *Journal of Child Psychology and Psychiatry*, *60*(1), 30-42.	Yes	Yes
