# Supplementary material for: Mental health resilience in offspring of depressed parents: a systematic literature review protocol
Source: Syst Rev. 2022 Sep 5;11:190. doi: 10.1186/s13643-022-02056-6 (PMC9446554; doi:10.1186/s13643-022-02056-6)
Supplement: Supplementary file 3 — Additional file 3. Full search strategy for each database [file 13643_2022_2056_MOESM3_ESM.pdf]

Mental health resilience in offspring of depressed parents: a systematic literature review

Electronic searches were performed on 15th March 2021.

Search strategies by database

Database: APA PsycInfo <1806 to March Week 2 2021>

Search Strategy:

----

1 exp Caregivers/ (29980)

2 exp Parents/ (120534)

3 exp Affective Disorders/ (149496)

4 exp Major Depression/ (136988)

5 exp Offspring/ (16831)

6 exp "Resilience (Psychological)"/ (15395)

7 exp Protective Factors/ (6050)

8 exp "Literature Review"/ (23361)

9 exp "Systematic Review"/ (545)

10 exp Meta Analysis/ (4924)

11 exp Qualitative Methods/ (16738)

12 (caregiver* or parent* or maternal or paternal or mother* or father*).ab,id,ot,ti. (432110)

13 (depress* or (affective adj2 disorder*) or (mood adj2 disorder*)).ab,id,ot,ti. (335164)

14 (offspring* or child* or son* or daughter*).ab,id,ot,ti. (775618)

15 (resilien* or protect* or buffer* or (mitigat* adj2 risk) or (optimi* adj2 outcome*) or (adapt* adj2 functioning) or thriv* or (positive adj2 adapt*)).ab,id,ot,ti. (157773)

16 (review* or comment* or letter or metaanalysis or meta-analysis or (meta adj2 analysis) or editorial or (conference adj2 publication) or qualitative).ab,id,ot,ti. (905449)

17 1 or 2 or 12 (438728)

18 3 or 4 or 13 (339691)

19 5 or 14 (776838)

20 6 or 7 or 15 (158549)

21 8 or 9 or 10 or 11 or 16 (910145)

22 17 and 18 and 19 and 20 (2444)

23 22 not 21 (2102)

****

Database: EMBASE <1947-Present>

Search Strategy:

----

1 exp caregiver/ (88034)

2 exp parent/ (292791)

3 exp depression/ (511079)

4 exp mood disorder/ (556439)

5 exp psychological resilience/ (4818)

6 exp child/ (3137100)

7 exp "review"/ (2801432)

8 exp "systematic review"/ (289282)

9 exp meta analysis/ (212275)

10 exp conference paper/ (1968054)

11 exp qualitative research/ (86495)

12 (caregiver* or parent* or maternal or paternal or mother* or father*).ab,kw,ot,ti. (1193552)

13 (depress* or (affective adj2 disorder*) or (mood adj2 disorder*)).ab,kw,ot,ti. (719616)

14 (offspring* or child* or son* or daughter*).ab,kw,ot,ti. (2425485)

15 (resilien* or protect* or buffer* or (mitigat* adj2 risk) or (optimi* adj2 outcome*) or (adapt* adj2 functioning) or thriv* or (positive adj2 adapt*)).ab,kw,ot,ti. (1385648)

16 (review* or comment* or letter or metaanalysis or meta-analysis or (meta adj2 analysis) or editorial or (conference adj2 publication) or qualitative).ab,kw,ot,ti. (3779086)

17 1 or 2 or 12 (1250118)

18 3 or 4 or 13 (907089)

19 5 or 15 (1386501)

20 6 or 14 (4037790)

21 7 or 8 or 9 or 10 or 11 or 16 (7049614)

22 17 and 18 and 19 and 20 (3181)

23 22 not 21 (2372)

****

Database: Ovid MEDLINE(R) ALL <1946 to March 14, 2021>

Search Strategy:

----

1 exp Parents/ (117476)

2 exp Caregivers/ (38846)

3 exp Mood Disorders/ (124775)

4 exp Depression/ (125189)

5 exp Child/ (1950649)

6 exp Resilience, Psychological/ (6546)

7 exp Protective Factors/ (4858)

8 exp "Systematic Review"/ (147716)

9 exp "Review"/ (2777286)

10 exp Meta-Analysis/ (128007)

11 exp Qualitative Research/ (61005)

12 (caregiver* or parent* or maternal or paternal or mother* or father*).ab,kw,ot,ti. (872245)

13 (depress* or (affective adj2 disorder*) or (mood adj2 disorder*)).ab,kw,ot,ti. (500597)

14 (offspring* or child* or son* or daughter*).ab,kw,ot,ti. (1705576)

15 (resilien* or protect* or buffer* or (mitigat* adj2 risk) or (optimi* adj2 outcome*) or (adapt* adj2 functioning) or thriv* or (positive adj2 adapt*)).ab,kw,ot,ti. (1047985)

16 (review* or comment* or letter or metaanalysis or meta-analysis or (meta adj2 analysis) or editorial or (conference adj2 publication) or qualitative).ab,kw,ot,ti. (2800834)

17 1 or 2 or 12 (903889)

18 3 or 4 or 13 (545540)

19 5 or 14 (2698778)

20 6 or 7 or 15 (1051349)

21 8 or 9 or 10 or 11 or 16 (4358303)

22 17 and 18 and 19 and 20 (1971)

23 22 not 21 (1665)

****

Database: Web of Science Core Collection

Search Strategy:

# 7	3,193	#6 NOT #5<br>*Indexes=SCI-EXPANDED, SSCI, A&HCI, CPCI-S, CPCI-SSH, ESCI Timespan=All years*
# 6	3,782	#4 AND #3 AND #2 AND #1<br>*Indexes=SCI-EXPANDED, SSCI, A&HCI, CPCI-S, CPCI-SSH, ESCI Timespan=All years*
# 5	4,109,336	TS= (review* or comment* or letter or metaanalysis or meta-analysis or (meta near/1 analysis) or editorial or (conference near/1 publication) or qualitative)<br>*Indexes=SCI-EXPANDED, SSCI, A&HCI, CPCI-S, CPCI-SSH, ESCI Timespan=All years*
# 4	1,874,835	TS=(resilien* or protect* or buffer* or (mitigat* near/1 risk) or (optimi* near/1 outcome*) or (adapt* near/1 functioning) or thriv* or (positive near/1 adapt*))<br>*Indexes=SCI-EXPANDED, SSCI, A&HCI, CPCI-S, CPCI-SSH, ESCI Timespan=All years*
# 3	2,611,007	TS=(offspring* or child* or son* or daughter*)<br>*Indexes=SCI-EXPANDED, SSCI, A&HCI, CPCI-S, CPCI-SSH, ESCI Timespan=All years*
# 2	682,647	TS=(depress* or (affective near/1 disorder*) or (mood near/1 disorder*))

		Indexes=SCI-EXPANDED, SSCI, A&HCI, CPCI-S, CPCI-SSH, ESCI Timespan=All years
# 1	1,131,940	TS=(caregiver* or parent* or maternal or paternal or mother* or father*) *Indexes=SCI-EXPANDED, SSCI, A&HCI, CPCI-S, CPCI-SSH, ESCI Timespan=All years*

Database: Cochrane Library

Search Strategy:

ID	Search	Hits
#1	MeSH descriptor: [Caregivers] explode all trees	2167
#2	MeSH descriptor: [Depression] explode all trees	12583
#3	MeSH descriptor: [Mood Disorders] explode all trees	13121
#4	MeSH descriptor: [Child] explode all trees	56688
#5	MeSH descriptor: [Resilience, Psychological] explode all trees	249
#6	MeSH descriptor: [Protective Factors] explode all trees	127
#7	MeSH descriptor: [Review] explode all trees	2
#8	MeSH descriptor: [Meta-Analysis as Topic] explode all trees	329
#9	MeSH descriptor: [Systematic Reviews as Topic] explode all trees	26
#10	MeSH descriptor: [Congresses as Topic] explode all trees	29
#11	MeSH descriptor: [Qualitative Research] explode all trees	1105
#12	((caregiver* or parent* or maternal or paternal or mother* or father*)):ti,ab,kw	84326
#13	((depress* or (affective near/1 disorder*) or (mood near/1 disorder*))):ti,ab,kw	90405
#14	((offspring* or child* or son* or daughter*)):ti,ab,kw	174791

#15 ((resilien* or protect* or buffer* or (mitigat* near/1 risk) or (optimi* near/1 outcome*) or (adapt* near/1 functioning) or thriv* or (positive near/1 adapt*))):ti,ab,kw 47840

#16 ((review* or comment* or letter or metaanalysis or meta-analysis or (meta near/1 analysis) or editorial or (conference near/1 publication) or qualitative)):ti,ab,kw 117477

#17 #1 OR #12 84326

#18 #2 OR #3 OR #13 90436

#19 #4 OR #14 174791

#20 #5 OR #6 OR #15 47840

#21 #7 OR #8 OR #9 OR #10 OR #11 OR #16 117499

#22 #17 AND #18 AND #19 AND #20 333

#23 #22 NOT #21 262
